# Susceptibility to ventilator induced lung injury is increased in senescent rats

**DOI:** 10.1186/cc12744

**Published:** 2013-05-27

**Authors:** Florian Setzer, Karsten Oschatz, Lars Hueter, Barbara Schmidt, Konrad Schwarzkopf, Torsten Schreiber

**Affiliations:** 1 Department of Anesthesiology and Intensive Care Medicine, Jena University Hospital, Erlanger Allee 101, 07747 Jena, Germany; 2 Department of Anesthesia and Intensive Care, Zentralklinik Bad Berka, Robert-Koch-Allee 9, 99437 Bad Berka, Germany; 3 Department of Anesthesia and Intensive Care, Klinikum Saarbrücken, Winterberg 1, 66119 Saarbrücken, Germany

**Keywords:** ventilator-induced lung injury, age, mechanical ventilation, tidal volume, volutrauma, inflammation, cytokines, lung lavage

## Abstract

**Introduction:**

The principal mechanisms of ventilator induced lung injury (VILI) have been investigated in numerous animal studies. However, prospective data on the effect of old age on VILI are limited. Under the hypothesis that susceptibility to VILI is increased in old age, we investigated the pulmonary and extrapulmonary effects of mechanical ventilation with high tidal volume (VT) in old compared to young adult animals.

**Interventions:**

Old (19.1 ± 3.0 months) and young adult (4.4 ± 1.3 months) male Wistar rats were anesthetized and mechanically ventilated (positive end-expiratory pressure 5 cmH2O, fraction of inspired oxygen 0.4, respiratory rate 40/minute) with a tidal volume (VT) of either 8, 16 or 24 ml/kg for four hours.

Respiratory and hemodynamic variables, including cardiac output, and markers of systemic inflammation were recorded throughout the ventilation period. Lung histology and wet-to-dry weight ratio, injury markers in lung lavage and respiratory system pressure-volume curves were assessed *post mortem*. Basic pulmonary characteristics were assessed in non-ventilated animals.

**Results:**

Compared to young adult animals, high VT (24 ml/kg body weight) caused more lung injury in old animals as indicated by decreased oxygenation (arterial oxygen tension (PaO2): 208 ± 3 vs. 131 ± 20 mmHg; *P *<0.05), increased lung wet-to-dry-weight ratio (5.61 ± 0.29 vs. 7.52 ± 0.27; *P *<0.05), lung lavage protein (206 ± 52 mg/l vs. 1,432 ± 101; *P *<0.05) and cytokine (IL-6: 856 ± 448 vs. 3,283 ± 943 pg/ml; *P *<0.05) concentration. In addition, old animals ventilated with high VT had more systemic inflammation than young animals (IL-1β: 149 ± 44 vs. 272 ± 36 pg/ml; *P *<0.05 - young vs. old, respectively).

**Conclusions:**

Ventilation with unphysiologically large tidal volumes is associated with more lung injury in old compared to young rats. Aggravated pulmonary and systemic inflammation is a key finding in old animals developing VILI.

## Introduction

Deleterious pulmonary effects of injurious ventilation strategies have been documented in numerous animal and clinical studies [1-3].

Many studies on ventilator induced lung injury (VILI) were performed in small animals, mostly rodents. These animals typically represent an adolescent or young adult population (for example, 8- to 16-week-old rats) [4-8] while the natural life span of most rats bred for research purposes is considerably longer [9,10]. In contrast, the majority of mechanically ventilated patients in adult intensive care units belong to the elderly population [11]. Ageing affects the function and the response to critical illness of many organs, including the respiratory [12,13] and immune systems [14,15] and clinical data indicate that, following pulmonary affections such as aspiration or pneumonia, outcome is worse in older than in younger patients [16,17].

In the light of such age-dependent alterations one may question if experimental data on VILI (a syndrome which in its evolution involves inflammatory processes and affects many aspects of lung physiology and function) derived from adolescent or young adult animals are representative for the elderly organism.

Knowledge on the effects of age on VILI is limited. Some studies in rats, focusing on age from the neonatal period to early adulthood, reported decreased [6,8], as well as increased [18], susceptibility to injurious ventilation of newborn and infant as compared to adult animals. These somewhat disparate results reflect effects of mechanical ventilation on immature, developing and mature, but not on aged lungs. Taking into account the different pulmonary physiology [7,19] and immune function [20] in elderly versus young mammals, it seems difficult to predict from these data the response to injurious ventilation in senescent subjects. To our knowledge, so far only one experimental study explored the effects of a brief, highly aggressive ventilation strategy in old rats and found aggravated lung injury in this population compared to young adult animals [21].

To further investigate if susceptibility to VILI is increased in old animals, we ventilated healthy rats of two different age groups with constant positive end-expiratory pressure (PEEP) but increasing tidal volume and assessed markers of inflammation and lung injury.

## Materials and methods

This study was approved by the institutional and local Committee on the Care and Use of Animals (Thüringer Landesamt für Lebensmittelsicherheit und Verbraucherschutz, Bad Langensalza, Germany; registration number 02-016/06). Male Wistar rats, from an in-house bred Wistar Han strain were used for the experiments.

### Age groups

Experiments were carried out in rats of two different age groups: Age group I (Young): young adult animals 4.4 ± 1.3 months old, weighing 378 ± 16 g. Age group II (Old): animals 19 ± 3.0 months old, weighing 583 ± 65 g.

### Series I: experiments in non-ventilated animals

A pilot study was performed in non-ventilated animals of both age groups to assess basic pulmonary variables. All animals were anesthetized with sodium thiopental (50 mg i.p.) and weighed.

In four animals of each age group an arterial blood sample was collected (while 100% oxygen was applied *via *a face-shoulder mask) for blood gas analysis and blood cell count. Median laparotomy was performed and animals exsanguinated by cannulating the inferior vena cava. Sternotomy was performed and lungs were harvested *en bloc *for further sampling. Post-mortem analyses included lung lavage, histology and wet-to-dry weight ratio.

In another four animals of each age group, after exsanguination, tracheostomy was performed and static pressure-volume curves of the respiratory system were obtained (*post mortem *measurements: see below).

The exposure time to anesthesia and 100% oxygen until non-ventilated animals were sacrificed was between 10 and 15 minutes.

### Series II: experiments in ventilated animals

Animals of both age groups were randomized to undergo mechanical ventilation with low (that is, protective) tidal volume (VT) of 8 ml/kg [22-24] or high or very high VT of 16 or 24 ml/kg body weight, respectively, for 4 hours (6 groups; *n = *15 per group).

### Instrumentation

After weighing, animals were anesthetized with sodium thiopental (50 mg i.p.) and procedural antibiotic prophylaxis with ceftriaxone 12.5 mg s.c. was applied. The right jugular vein and carotid artery were surgically exposed, and catheters for fluid infusion and blood pressure monitoring inserted. A thermistor probe (Microprobe F1.5, Columbus Instruments, Columbus, OH, USA) was advanced to the aortic arch via the left carotid artery and connected to a cardiac output (CO) computer (Cardiotherm 400R, Columbus Instruments). Tracheostomy was performed and a blunt cannula (outer diameter 2 mm) was tightly secured in the tracheal lumen.

Mechanical ventilation (MV) using a fraction of inspired oxygen = 0.4, respiratory rate = 40 breaths/minute, inspiration-to-expiration ratio = 1, PEEP = 5 cm H2O was started (Animal Respirator CIV 101, Columbus Instruments).

According to randomization, VT was adjusted to 8, 16 or 24 ml/kg body weight, respectively. In old animals weighing 550 g or less, VT was calculated according to actual body weight. In previous experiments we did not see an increase in lung weight in animals exceeding 550 g body weight. Hence, in animals exceeding 550 g body weight, for calculation of VT, a body weight of 550 g was assumed.

In animals ventilated with VT of 16 or 24 ml/kg, CO2 was added to the inspiratory gas if required, to maintain arterial normocapnia and prevent respiratory alkalosis while respiratory rate was unchanged.

Pancuronium bromide 0.5 mg was injected intravenously for muscle relaxation after the beginning of MV and a continuous infusion of balanced electrolyte solutions at a rate of 10 ml/h (Thomaejonin, Delta Pharma GmbH, Pfullingen Germany) was started. Airway pressures were recorded, using a side port of the tracheal cannula. Anesthesia was maintained through intravenous infusion of ketamine and midazolam. A body temperature of 38°C was maintained throughout the experiment using a closed-loop warming system (HSE-Temperaturregler, Hugo Sachs Elektronik, March, Germany).

### Ventilation period/*in vivo *measurements

During the ventilation period, hemodynamic and respiratory variables, including arterial blood gases, were recorded hourly. Thermodilution cardiac output was determined at the beginning, at 2 h and at 4 h of MV by central-venous injection of 100 μl ice-cooled isotonic saline. The mean of three repeated injections at each time point was recorded. Blood was drawn for blood cell count and plasma cytokine assessment at the beginning, at 2 h and at 4 h of MV. Total white blood cell counts (TWBC) were determined with a hemocytometer. Percentage of neutrophils was obtained from a cell smear (May-Gruenwald-Giemsa stain; 100 white blood cells counted at a magnification of x500) and absolute neutrophil numbers were calculated according to the TWBC.

After four hours of MV, animals were exsanguinated by cannulating the inferior vena cava.

### Post mortem measurements

Animals surviving less than three hours of MV were *a priori *excluded from *post mortem *data analysis. This was done to avoid pooling results reflecting very different time points in case of markers which are known to be expressed time dependently (such as, for example, cytokines and cell counts in lung lavage).

#### Lung static pressure-volume curves

Lungs were manually inflated with a 20 ml syringe connected to the tracheal cannula to a pressure of 25 cm H2O which was held for 5 s and then allowed to deflate passively. Lungs were then stepwise inflated in increments of 1 ml to a maximum volume of 15 ml and subsequently deflated in steps of 1 ml. Airway pressure was recorded with each step.

#### Lung weight

Sternotomy was performed, lungs were removed *en bloc *from the thorax, left and right lungs weighed separately and then processed for further sampling.

#### Lung lavage

Left lungs were lavaged with 10 ml phosphate buffered saline as previously described [25] and the effluents centrifuged at 3,000 rpm for 10 minutes. Neutrophils and macrophages in the resulting cell pellet were counted using a hemocytometer and numbers corrected for the total amount of bronchoalveolar lavage fluid.

Protein content in lavage fluid supernatant was measured using turbidimetry (assay: Roche Diagnostics, Roche Deutschland Holding GmbH, Grenzach-Wyhlen, Germany; analyzer: Hitachi 717, Boehringer Mannheim, Mannheim, Germany ) with a detection threshold of 60 mg/l.

#### Lung wet-to-dry weight ratio (WD ratio)

The cranial (that is, superior) lobe of the right lung was weighed, desiccated at 48°C for 48 h (heating oven: TH15, Edmund Bühler, Tübingen, Germany), weighed again, and WD ratio was calculated.

#### Lung histology

The remaining three lobes of right lungs were formalin fixed, paraffin embedded and sagittally cut to obtain 5 µm thick slices. Hematoxylin and eosin-stained slides were prepared for light microscopy. In 10 randomly selected high power fields (magnification x400) per slide (excluding fields containing conducting airways and blood vessels) the number of alveolar neutrophils and the percentage of alveoli, alveolar sacs and alveolar ducts containing any hyaline membranes/azidophilic material were recorded [26]. The investigator (BS) was blinded as to group allocation of the tissue section.

#### Cytokine levels in plasma and supernatant of lung lavage

Cytokine levels in lung lavage fluid supernatant and blood plasma were assessed with commercially available ELISA kits specific for rat (KRC0062 (IL-6), KRC00011 (IL-1β), KRC1022 (MIP-2α); BioSource, Solingen, Germany) used according to the manufacturer's guidelines.

### Statistical analysis

Data are presented as mean ± SEM. Analysis of variance with the factors age and VT was used to identify between group differences in lung injury markers. Analysis of variance using a repeated measures term was performed for comparison of variables between groups over time (cardio-respiratory variables, blood neutrophil counts, plasma cytokine levels). Tukey B correction was used for *post hoc *analysis in case of multiple comparisons. Chi-square test was used to analyze differences in mortality between groups. *P *<0.05 was considered statistically significant. The statistics software IBM SPSS Statistics, Version 19, IBM Corporation, Armonk, New York, United States was used for data analysis.

## Results

### Non-ventilated animals (Table 1 and Figure 1)

**Figure 1:**
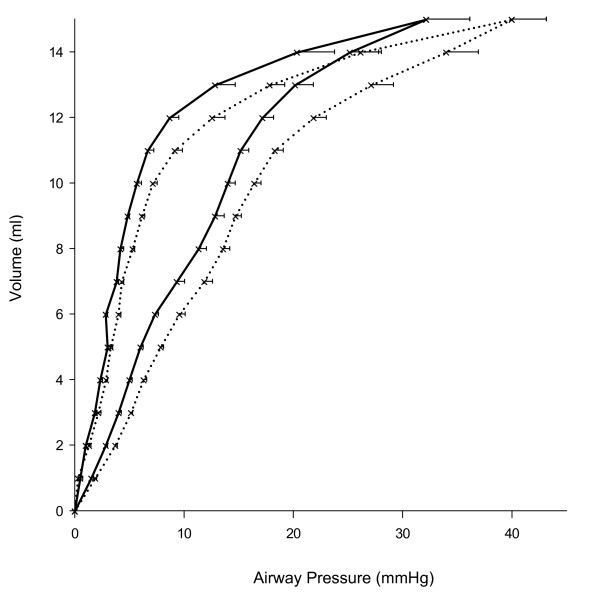
**Respiratory system static pressure-volume curves obtained from non-ventilated rats**. Young animals (*n *= 4): dotted lines; old animals (*n *= 4): solid lines..

In non-ventilated rats there was no difference between the two age groups regarding lung WD ratio, lung lavage macrophages and protein content. Neutrophils in lung lavage were detectable in none of the young and in low numbers in only one old animal. In TWBC, but not in blood neutrophil counts, there was a significant difference between age groups. Arterial oxygen tension(measured while fraction of inspired oxygen 1.0 was applied) was significantly lower in old than in young animals. Static pressure volume curves of the respiratory system were shifted to the left in old rats indicating better respiratory system compliance in this group (Figure 1).

**Table 1:** White blood cell counts and pulmonary variables in non-ventilated old and non-ventilated young rats (*n *= 4/group).

	Old rats	Young rats	*P*
TWBC (/μl)	4,800 ± 524	9,037 ± 638	0.02
_PMN in blood (/μl)	867 ± 146	1,162 ± 65	Ns
_Macrophages in BAL (/μl)	11,744 ± 2,172	10,882 ± 2,646	Ns
_Protein in BAL (mg/l)	69 ± 35	97 ± 30	Ns
_Lung wet/dry weight ratio	4.68 ± 0.09	4.65 ± 0.06	Ns
_PaO2 at FiO2 1.0 (mmHg)	270 ± 60	446 ± 10	0.029
_PMN in BAL (/μl)	Not detectable in 3; low counts in 1 animal	Not detectable	n.a.

Values are mean ± SEM.

BAL, bronchoalveolar lavage; FiO2, fraction of inspired oxygen; n.a., not applicable; PaO2, arterial oxygen tension; PMN, polymorphonuclear neutrophils; TWBC, total white blood cell count

### Ventilated animals

#### Hemodynamic and respiratory parameters

**Figure 2:**
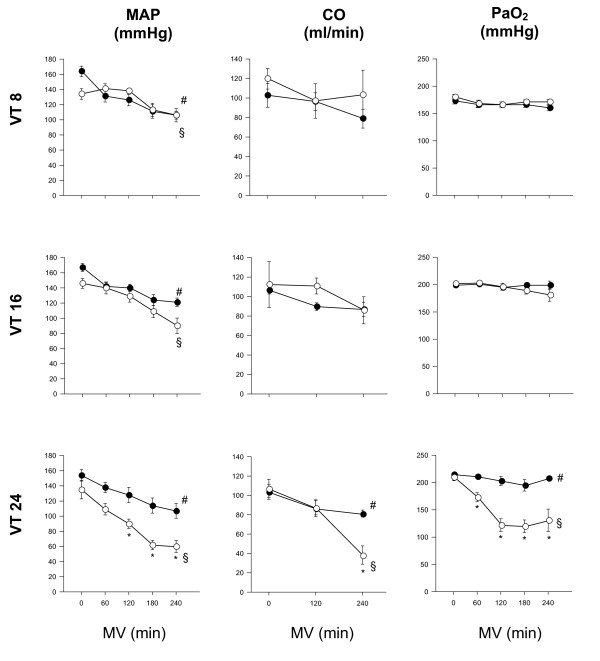
**Cardio-respiratory parameters in old and young mechanically ventilated rats**. Mean arterial pressure (MAP), cardiac output (CO) and arterial oxygen tension (PaO2) in old (white circles) versus young (black circles) rats in minutes after initiation of mechanical ventilation (MV) with tidal volume (VT) of 8, 16 or 24 ml/kg body weight, respectively. * = *P *<0.05 old vs. young animals; #, § = *P *<0.05 time point vs. start of MV.

**Figure 3:**
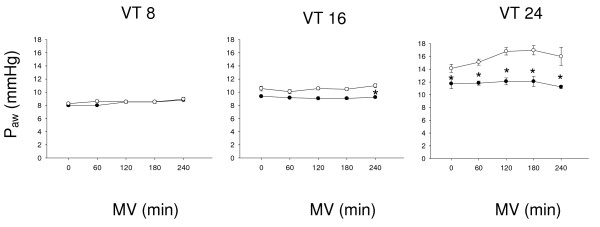
**Mean airway pressures (Paw) in young (black circles) versus old (white circles) mechanically ventilated rats**. Mechanical ventilation (MV) was applied with tidal volume (VT) of 8, 16 or 24 ml/kg body weight, respectively. * = *P *<0.05 old vs. young animals; #, § = *P *<0.05 time point vs. start of MV.

There was a decrease in mean arterial pressure at the end of MV in all groups. This was most pronounced in old animals ventilated with VT 24 ml/kg (Figure 2). Cardiac output remained constant during MV with 8 or 16 ml/kg but decreased in animals ventilated with 24 ml/kg and significantly stronger in old than in young animals (Figure 2). PaO_2 _was comparable and constant during MV with 8 or 16 ml/kg in both age groups and remained stable in young but decreased significantly in old animals ventilated with 24 ml/kg (Figure 2). Mean airway pressures were similar in both age groups during MV with 8 or 16 ml/kg, but significantly higher in old than in young animals during MV with 24 ml/kg (Figure 3). Further cardio-respiratory parameters are shown in Table 2.

**Table 2:** Cardio-respiratory parameters in old and young mechanically ventilated rats

	VT 8	VT 8	VT 16	VT 16	VT 24	VT 24
	**beginning**	**end**	**beginning**	**end**	**Beginning**	**end**

_pH						
old	7.44 (± 0.19)	7.42 (± 0.02)	7.39 (± 0.01)	7.36 (± 0.03)	7.41 (± 0.18)	7.24 (± 0.06)
young	7.36 (± 0.02)	7.34 (± 0.01)	7.44 (± 0.19)	7.43 (± 0.13)	7.40 (± 0.01)	7.46 (± 0.02)

_paCO2 (mmHg)						
old	38.8 (± 2.1)	35.3 (± 2.6)	37.8 (± 1.5)	36.7 (± 1.1)	36.4 (± 0.9	34.8 (± 1.3)
young	47.0 (± 1.9)	48.7 (± 1.5)	38.2 (± 1.6)	38.7 (± 1.0)	40.1 (± 1.2)	38.1 (± 1.0)

_CVP (mmHg)						
Old	5.1 (± 0.6)	5.0 (± 0.5)	5.9 (± 0.6)	5.3 (± 0.6)	5.1 (± 0.3)	6.0 (±0.7)
young	4.9 (± 0.5)	4.3 (± 0.5)	5.3 (± 0.6)	5.1 (± 0.6)	6.9 (± 0.6)	5.9 (± 0.5)

_Heart rate (bpm)						
old	397 (± 11)	426 (± 15)	413 (± 10)	390 (± 23)	380 (± 16)	415 (±10)
young	432 (± 15)	436 (± 8)	448 (± 7)	439 (± 9)	421 (± 7)	418 (± 9)

_Parameters were assessed at the beginning and at the end of the mechanical ventilation period. Tidal volumes (VT) were 8, 16 or 24 ml/kg, respectively.

#### Respiratory system static pressure volume curves

**Figure 4:**
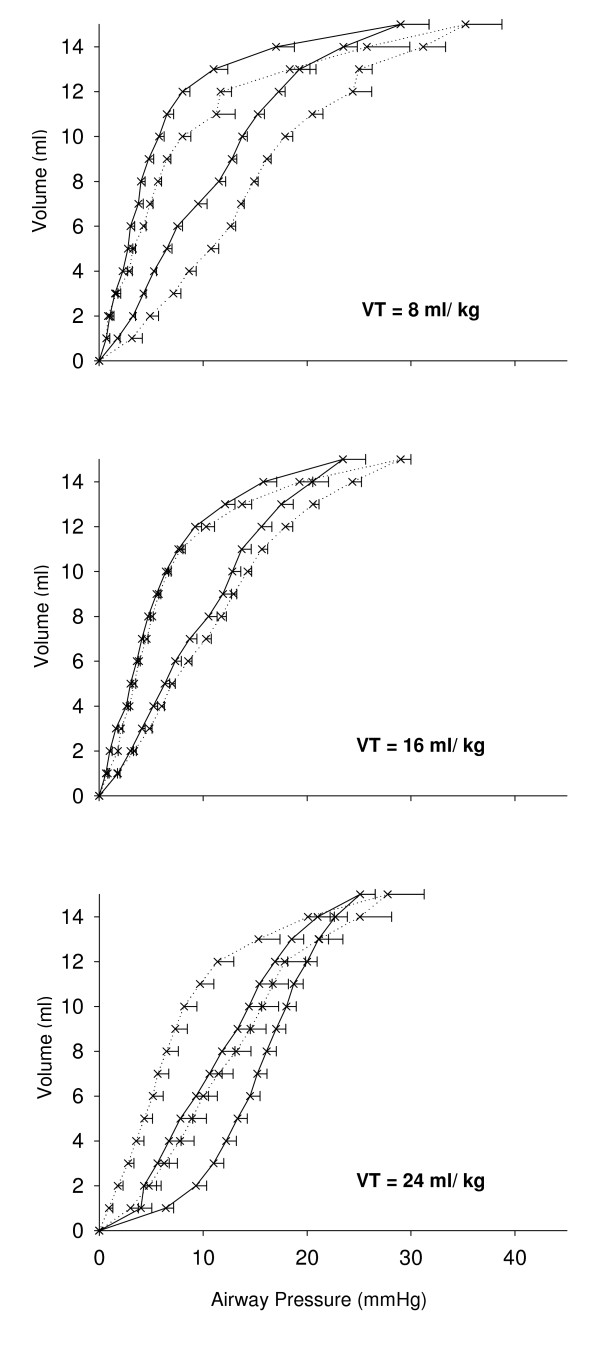
**Respiratory system static pressure-volume curves in ventilated young (dotted lines) and old (solid lines) rats**. Mechanical ventilation (MV) was applied for 4 hours with tidal volume (VT) of 8, 16 or 24 ml/kg body weight, respectively. The shift of the curves is significant in animals ventilated with VT 8 and 24 ml/kg body weight, respectively.

While following ventilation with VT 8 ml/kg compliance is significantly better in old animals (as is the case in non-ventilated animals), the opposite is true after ventilation with VT 24 ml/kg (Figure 4).

#### Survival

In comparison to young animals, mortality before the end of the ventilation period was significantly increased in old animals ventilated with VT 24 ml/kg (Table 3).

**Table 3:** Survival in old and young mechanically ventilated rats

Survival	>3 hours of MV		Complete ventilation period (4 h)	
**_Age**	**young**	**old**	**young**	**old**

_VT 8 ml/kg	100%	100%	87%	93%
_VT 16 ml/kg	100%	87%	100%	80%
_**VT 24 ml/kg**	**93%**	**73%**	**80%**	33% *

N = 15 per group;

Animals were ventilated with either low (8 ml/kg), high (16 ml/kg) or very high (24 ml/kg) tidal volume (VT). * *P *= 0.013 vs. old animals with same tidal volume at the same time point.

#### Lung WD ratio

**Figure 5:**
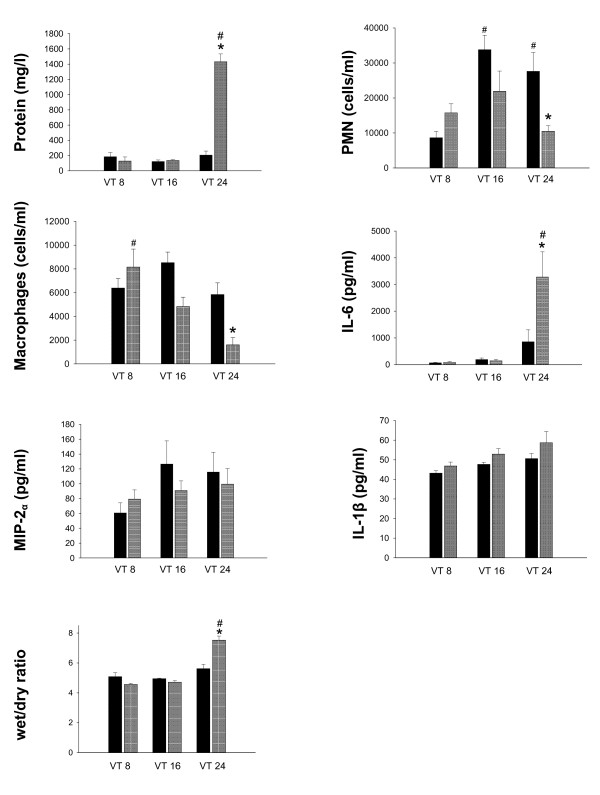
**Lung inflammation markers and wet-to-dry weight ratio in young versus old mechanically ventilated rats**. Protein content, neutrophil count (PMN), macrophage count (macrophages), interleukin-6 (IL-6), macrophage inhibitory protein 2 (MIP-2α) and interleukin-1β (IL-1β) in lung lavage fluid and lung wet-to-dry weight ratio (WD ratio) in old (gray bars) and young rats (black bars). Animals were ventilated for 4 hours with tidal volume (VT) of 8, 16 or 24 ml/kg body weight, respectively. * = *P *<0.05 young vs. old animals with same VT; # = *P *<0.05 versus VT 8 in same age group.

There was a significant increase in lung WD ratio in old animals ventilated with VT 24 ml/kg compared to the same VT in young animals and compared to old animals ventilated with 8 ml/kg (Figure 5).

#### Lung lavage

*Protein content: *There was no effect of age on lung lavage protein content in animals ventilated with VT 8 and 16 ml/kg. However, protein content was significantly increased in old compared to young animals ventilated with 24 ml/kg and compared to old animals ventilated with 8 ml/kg (Figure 5). *Neutrophil counts: *In young, but not in old animals, there was a significant increase in lung lavage neutrophils with increased VT. After MV with VT 24 ml/kg neutrophil counts were significantly higher in young than in old animals (Figure 5). *Macrophage counts: *Macrophage numbers in lung lavage decreased with increasing VT in old but not in young animals. After ventilation with VT 24 ml/kg macrophage numbers were lower in old than in young animals (Figure 5). *Cytokines: *IL-6 levels in lung lavage fluid were significantly higher in old compared to young animals ventilated with 24 ml/kg (Figure 5). There were no significant effects of age or VT regarding levels of MIP-2 in lung lavage fluid (Figure 5). IL-1β in lung lavage increased with VT, but not differently so between age groups (Figure 5).

#### Lung histology

**Figure 6:**
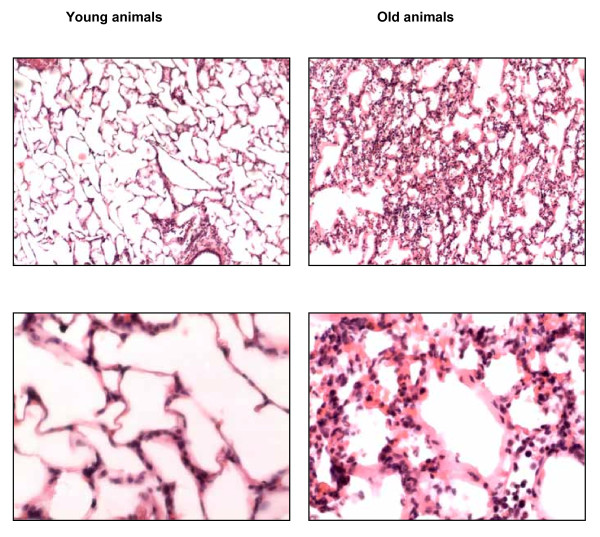
**Representative lung histology in young and old rats follwing mechanical ventilation with high tidal volume**. Tidal volume was 24 ml/kg for 4 h. Tissue sections are stained with hematoxylin and eosin. Magnification is 100x (upper panel) and 400x (lower panel).

Irrespective of VT, numbers of neutrophils in airspace and the proportion of airspace containing hyaline membranes were higher in old than in young animals (data not shown). Representative tissue sections are shown in Figure 6.

#### Systemic inflammation

**Figure 7:**
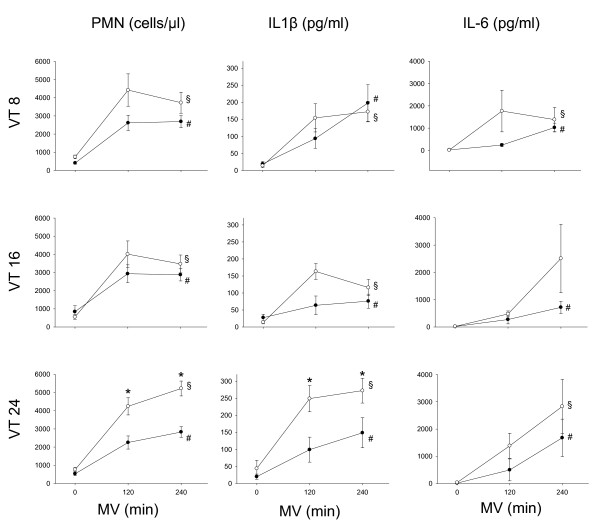
**Markers of systemic inflammation during mechanical ventilation (MV) in young and old rats**. Young (black circles) and old (white circles) rats were ventilated with tidal volumes (VT) of 8, 16 or 24 ml/kg, respectively. PMN, blood neutrophil counts; IL-1β, Interleukin-1β in serum; IL-6, Interleukin-6 in serum. *, *P *<0.05 old vs. young animals; #, §, *P *<0.05 time point vs. start of MV.

Blood neutrophil counts and IL-1β in serum (Figure 7) significantly increased at the end of the ventilation period in all groups. According to age there was no difference in these parameters during MV with VT 8 or 16 ml/kg but they both were significantly higher in old than in young animals ventilated with VT 24 ml/kg.

Regarding IL-6 in serum, there was an increase at the end of MV in all groups (except in old animals with VT 16 ml/kg) but without differences regarding age (Figure 7).

## Discussion

The principal finding of our *in vivo *study is that old animals are more susceptible to injurious high tidal volume ventilation than young adult animals.

Following ventilation with low and moderately increased VT, differences seen were an increased number of intrapulmonary neutrophils and more hyaline membranes in old compared to young animals. Following ventilation with the highest VT, several aspects of lung injury were present in the lungs of old but not of young animals. Findings include increased pulmonary edema, inflammation and attenuated lung function. In addition, in old animals the highest VT was associated with more extra-pulmonary adverse effects compared to young animals, as indicated by pronounced hemodynamic depression, an accentuated systemic pro-inflammatory response and higher mortality during the ventilation period.

Using low or so-called protective VT [22-24] as a control, we applied two levels of increased VT to provoke lung injury. Supraphysiologic tidal volumes are often used to investigate lung injury in *in vivo *ventilated small animals. This approach reflects the compromise between limiting the ventilation period to a few hours (in order to limit the role of confounding factors, such as conduct of anesthesia, fluid status, risk of infection, nutrition and so on) and the attempt to produce detectable lung injury in this short time.

The highest VT level was selected according to previous experiments, revealing that 24 ml/kg applied for four hours caused some injury but was not associated with significant mortality in young adult rats [27]. We suspected, however, that in old rats such VT might be less well tolerated. It was thus decided to include a group ventilated with moderately increased VT, an approach that also offered the prospect of identifying a possible dose-related response to VT.

The present study underscores the potential of injurious ventilation to elicit an inflammatory response both locally (that is, in the lung) and systemically, an effect repeatedly shown in young adult animals [4,28-30]. Triggers of inflammation have been found to cause stronger responses in aged subjects [16,17]. Age-related alterations in lung immune function could possibly result in a lower threshold for a given inflammatory stimulus (such as increased lung distention) to translate to functional impairment. In conjunction with the effects seen on pulmonary and hemodynamic function, the inflammatory findings in our study support this theory.

The pronounced pro-inflammatory response in old animals is indicated by an increase in pulmonary and systemic cytokine levels, as well as by cellular effects. Similar to the effect seen in our old animal population, decreased macrophage numbers in lung lavage fluid, have previously been reported in young adult animals following injurious ventilation [4,24,27]. Although decreased by number in airspace, these cells were activated and contributed to lung injury [4,24], possibly by playing a role in the evolution of alveolo-capillary barrier breakdown. This, in consequence, could result in increased lung wet-to-dry weight ratio, as also found in the present experiment.

Of note, the effects of injurious ventilation on pro-inflammatory markers are not uniform in our study as revealed by lung lavage concentrations of MIP-2 and IL-1β. Differential kinetics of pulmonary cytokine expression has been discussed as one possible reason for such findings [31,32]. Further, we did not see a clear age-related effect of mechanical ventilation on neutrophils in lung lavage fluid, although, as assessed by light microscopy, more of these cells were present in the air space in aged lungs, irrespective of the VT applied. We speculate that factors involved in the different steps of leukocyte migration may have been differently expressed or activated in the different age groups. Neutrophil adhesion molecules have been shown to be differently expressed depending on the ventilation strategy applied [33]. In theory, such an effect, if also age dependent, could affect cell retrieval by lavage and thus play a role regarding these disparate findings.

In studies on VILI, aggressive ventilation strategies were frequently associated with arterial hypotension [29,30].

Arterial hypotension and decreased cardiac output occurred in old animals subjected to high VT in our study. Besides a possible role of different intravascular volume status (although the comparable central venous pressures in both age groups are not suggestive of that), systemic inflammation is a possible reason for this finding: IL-1β concentrations were highest in the animal group exhibiting the strongest hemodynamic depression (old animals ventilated with the highest VT) and an increase in IL-1β blood concentrations has been shown to induce arterial hypotension and cardiac depression in both rodents and humans [34-36]. In addition, in this group mortality was highest and animals which died prematurely uniformly developed severe and progressive hypotension and eventually acidosis and hypoxemia.

For the interpretation of the effect of MV on respiratory system, pressure-volume curves findings from non-ventilated animals are helpful. Respiratory system compliance was better in non-ventilated old animals, a finding also reported by others. Loss of elastic tissue recoil and changes in the size of terminal air spaces with increasing age are possible explanations [37]. While this difference between age groups was preserved following MV with low VT, increasing VT had a detrimental effect in old animals as indicated by the rightward shift of the curves.

While the question if effects of injurious ventilation are age-dependent was investigated in some studies focusing on animals from the neonatal period to early adulthood [6,8,18] data from aged animals are scarce. One recent study by Nin and co-workers compared the effects in young and elderly rats (*n *= 4 per group) of a highly aggressive ventilation protocol (VT 35 ml/kg) used over a brief period of time (60 minutes) [21]. The array of pulmonary parameters investigated was limited and lacked markers of inflammation, but histologic lung injury and the increase in airway pressure were aggravated in old animals.

Our findings add to these data by reporting a wider portfolio of markers of pulmonary injury and by extending the ventilation period to four hours, they give some insight in time-dependent evolution of injurious ventilation effects. In addition, while in the study by Nin *et al*. a combination of very high VT and no PEEP was used, we applied PEEP to all animals. Thus, our results reflect to a lesser extent a mixture of pulmonary over-inflation and atelectrauma, but may more specifically be related to volutrauma.

Besides an age-dependent expression of the inflammatory response, other age-dependent pulmonary characteristics could be considered as possible reasons for increased VILI in old animals. A given ventilation strategy could cause a different response to lung stretch due to differences, for example, in surfactant function or could translate to a more pronounced impairment in oxygenation because of an already decreased baseline functional reserve in higher age. However, given the paucity of data from ventilation studies including old animals, these mechanisms currently remain speculative.

Our study has some limitations. First, although we attempted to adjust the tidal volumes applied in both age groups to lung size, we cannot prove that we achieved a similar level of lung distension in both age groups with increasing VT. Total tidal volumes (that is, VT in ml per animal) differed by approximately 30% between old and young animals at each level of VT (in ml/kg body weight). While airway pressures were similar between age groups when low and moderately increased VT was applied, they were higher in old than in young animals from the beginning of MV with the highest VT. While the size of the tracheal cannula (which was the same in all animals) may have contributed to this effect, we also cannot exclude that in this group more lung distension may have occurred, translating to more pulmonary inflammation and injury.

Second, regarding age, our old animal population was not absolutely homogeneous. In previous observations in the same rat strain, we noticed spontaneous deaths if animals reached an age of 22 months and beyond. In order not to lose too many animals prior to the study period and not to accidentally include animals with imminent death, we chose to include animals of an age of >15 but <23 months. Still, from our own observation and data from the literature [9,10], we assume that animals in our study truly represent an ageing population.

Third, the same protocol for fluid and anesthetic infusion was used in both age groups. Thus, if possible age-specific drug requirements were missed, there is a risk that this affected our results. However, the similar hemodynamic conditions in both age groups during ventilation with low or moderately increased VT do not suggest a major role of such an effect. In addition, given the lack of recommendations for dosing of anesthetics and intravenous fluid in rats of different ages, it remains speculative if a different experimental protocol (for example, including a lower dose of anesthetics in old rats) could have avoided this shortcoming.

Further, to avoid hypocapnia resulting from ventilation with high VT a reduced respiratory rate (instead of adding CO2 at a constant respiratory rate) could have been applied. However, this approach bears the risk of obscuring the results. In case of no measurable injurious effect of high VT, it would have remained unclear if this is due to a protective effect of low respiratory rate counterbalancing the injurious effect of high VT, or if there truly is no effect of high VT.

Last, while effects of age on pulmonary function and physiology have been documented in rodents and humans [7,19], we are not aware of studies comparing, for example, the time course and the intensity of such age related changes between species. Hence, it remains somewhat unclear which particular age periods in human beings are reflected by our rat study. For clarification, this topic warrants further research.

## Conclusions

In conclusion, our study demonstrates pronounced VILI, systemic inflammation and hemodynamic depression in old rats ventilated with high VT, suggesting a greater susceptibility to volutrauma in this age group. Our findings are relevant with regard to both the experimental and clinical setting. In conjunction with previous data [21], they confirm in old animals the principal findings of VILI demonstrated in numerous experiments conducted in adolescent or young adult animals. Further, albeit keeping in mind that extrapolation of animal data to a clinical scenario has significant limitations, it is unlikely that in elderly humans the response to injurious ventilation is completely different from rodents. Thus the findings of this study suggest carefully adjusted ventilator settings in elderly patients, because with a given ventilation strategy the lungs of aged individuals may be more easily harmed than those of younger patients.

## Key messages

• Old as compared to young adult rats are more susceptible to VILI.

• The features of VILI in old rats are similar to the findings known from young adult rats.

## Abbreviations

CO: cardiac output; CVP: central venous pressure; FiO2: fraction of inspired oxygen; IL-1β: Interleukin 1β; IL-6: interleukin 6; MAP: mean arterial pressure; MIP 2: Macrophage inflammatory protein; MV: mechanical ventilation; PaO2: arterial oxygen tension; PEEP: positive end-expiratory pressure; TWBC: total white blood cell count; VILI: ventilator induced lung injury; VT: tidal volume; WD ratio: wet-to-dry weight ratio

## Competing interests

The authors declare that they have no competing interests.

## Authors' contributions

FS designed the experimental set-up, performed experiments, analyzed the results and drafted the manuscript. KO performed experiments and analyzed the results. LH helped analyze the results. BS helped design the experimental set-up and performed experiments. KS helped design the study and analyze the data. TS designed the study, helped analyze the data and revised the manuscript. All authors read and approved the final manuscript.
